# Antimicrobial Resistance in Africa: Public Health Challenges and Lessons From Morocco

**DOI:** 10.7759/cureus.111532

**Published:** 2026-06-26

**Authors:** Nadia Benaicha, Maryame El Khayari, Fatimazahrae Melki, Khaoula Achir, Hbibi Mohamed

**Affiliations:** 1 Department of Public Health, Faculty of Medicine Errachidia, Moulay Ismail University of Meknes, Errachidia, MAR; 2 Department of Gastroenterology and Hepatology, Faculty of Medicine Errachidia, Moulay Ismail University of Meknes, Errachidia, MAR; 3 Department of Endocrinology, Diabetology, Metabolic Diseases, and Nutrition, Faculty of Medicine Errachidia, Moulay Ismail University of Meknes, Errachidia, MAR; 4 Department of Pediatrics, Faculty of Medicine Errachidia, Moulay Ismail University of Meknes, Errachidia, MAR

**Keywords:** africa, antimicrobial resistance, antimicrobial stewardship, morocco, surveillance

## Abstract

Antimicrobial resistance (AMR) is recognized as one of the leading global public health threats, with a disproportionate impact on low- and middle-income countries, particularly in Africa, where high infectious disease burden, inappropriate antibiotic use, limited diagnostic capacity, and heterogeneous surveillance systems contribute to the emergence and spread of resistant pathogens. This narrative review synthesizes current evidence on AMR in Africa while highlighting Morocco as a relevant case study to inform public health priorities. A comprehensive literature search was conducted using PubMed/MEDLINE, Scopus, and Google Scholar, complemented by grey literature and World Health Organization (WHO) reports. Evidence published between 2014 and 2025 addressing AMR, antibiotic use, surveillance, stewardship, and policy interventions in Africa was narratively synthesized. Available evidence indicates that in 2019, approximately 4.95 million deaths were associated with bacterial AMR globally, including 1.27 million deaths directly attributable to AMR, with the highest mortality rates reported in western Sub-Saharan Africa. Across African settings, AMR is driven by nonprescription antibiotic access, self-medication, empirical broad-spectrum prescribing, weak infection prevention and control measures, and insufficient microbiological diagnostics, while surveillance remains fragmented because of uneven implementation of the WHO-Global Antimicrobial Resistance and Use Surveillance System (GLASS) recommendations. Morocco reflects many of these continental challenges, including high resistance among WHO priority pathogens such as *Escherichia coli *and *Acinetobacter baumannii*. Strengthening laboratory capacity, standardized surveillance, antimicrobial stewardship, and coordinated One Health policies remains essential to preserve the effectiveness of existing antibiotics in Africa.

## Introduction and background

Antimicrobial resistance (AMR) has emerged as one of the most critical threats to global public health, compromising the effectiveness of antimicrobial agents and threatening the foundations of modern medicine [1]. By reducing the efficacy of standard treatments, AMR contributes to therapeutic failure, prolonged hospital stays, increased healthcare costs, and avoidable mortality [2,3]. The impact of AMR is particularly concerning in low- and middle-income countries, where infectious disease burden remains high, and health systems often face limitations in diagnostic capacity, infection prevention and control, and access to appropriate antimicrobial therapy [4,5].

Importantly, the highest all-age mortality rates attributable to AMR have been reported in western sub-Saharan Africa, highlighting the disproportionate burden carried by African populations and the urgent need for regionally adapted public health responses [2]. In addition, systematic evidence from sub-Saharan Africa has documented widespread resistance among clinically relevant bacterial pathogens and highlighted important gaps in microbiological surveillance and laboratory capacity across the region [6]. Recent global analyses further suggest that AMR-related mortality could increase substantially by 2050 if effective control measures are not implemented [7].

In Africa, AMR is driven by a combination of community-level and healthcare-associated factors. Nonprescription access to antibiotics, self-medication, incomplete treatment courses, empirical broad-spectrum prescribing, and weak antimicrobial stewardship programs contribute to inappropriate antimicrobial exposure and selection pressure [8-11]. At the same time, limited microbiological laboratory infrastructure, insufficient antimicrobial susceptibility testing (AST), and fragmented surveillance systems restrict the ability of health systems to detect, monitor, and respond effectively to resistant pathogens [12-15].

Surveillance remains a cornerstone of AMR control, yet implementation across African countries remains heterogeneous. Studies evaluating progress in the implementation of the World Health Organization (WHO)-Global Antimicrobial Resistance and Use Surveillance System (GLASS) recommendations have shown persistent challenges in laboratory capacity, standardized reporting, quality assurance, and integration of surveillance data into national action plans [16-18]. These gaps limit comparability between countries and reduce the ability of policymakers to design evidence-based interventions tailored to local resistance patterns [16-18].

The One Health approach is increasingly recognized as essential for addressing AMR because resistant bacteria and resistance genes circulate across humans, animals, food systems, agriculture, and the environment [19,20]. In African settings, where human, animal, and environmental health systems are closely interconnected, multisectoral governance is particularly important to reduce unnecessary antimicrobial use and limit the spread of resistant organisms [8,16].

Morocco provides a relevant North African case study that reflects many of the broader AMR challenges observed across the continent. Published Moroccan studies have documented resistance among WHO priority pathogens, including multidrug-resistant *Escherichia coli*, extended-spectrum beta-lactamase (ESBL)-producing *Enterobacteriaceae*, *Klebsiella pneumoniae*, and carbapenem-resistant *Acinetobacter baumannii* [21-25]. The scoping review by Nejjari et al. synthesized available evidence on GLASS pathogens in Morocco and highlighted substantial resistance levels among key bacterial species, reinforcing the need to strengthen surveillance, stewardship, and infection prevention and control strategies [4].

This narrative review aims to synthesize current evidence on AMR in Africa, describe key determinants and surveillance challenges, and highlight Morocco as a case study to inform public health priorities. It also discusses antimicrobial stewardship, laboratory strengthening, infection prevention and control, and One Health strategies as essential components of a coordinated AMR response in African health systems.

## Review

Materials and methods

Study Design

This manuscript was designed as a narrative review aiming to provide a comprehensive public health overview of AMR in Africa while highlighting Morocco as an illustrative case study reflecting several AMR challenges observed across African health systems. The review was developed to synthesize available evidence regarding AMR burden, resistance patterns, surveillance systems, antimicrobial stewardship, infection prevention and control (IPC), and policy responses in African settings. In addition, the Moroccan component of this review was informed by a previously published epidemiological scoping review conducted by Nejjari et al., which synthesized resistance patterns among the WHO priority pathogens in Morocco and provided one of the most comprehensive mappings of published AMR evidence currently available in the country [4]. Morocco was selected as an illustrative case study because of the availability of published evidence, including a comprehensive scoping review of GLASS pathogens, its relevance within the North African context, and the opportunity to discuss AMR surveillance, stewardship, and policy challenges using a well-documented national example.

Because AMR in Africa remains influenced by complex interactions involving healthcare systems, antibiotic consumption, laboratory capacity, socioeconomic determinants, and public health governance, we intentionally selected a narrative review methodology rather than a strictly systematic review design. This approach allowed integration of epidemiological evidence, international surveillance reports, stewardship recommendations, and policy-oriented literature to better contextualize AMR challenges across heterogeneous African settings [2-7].

Search Strategy

**Figure 1:**
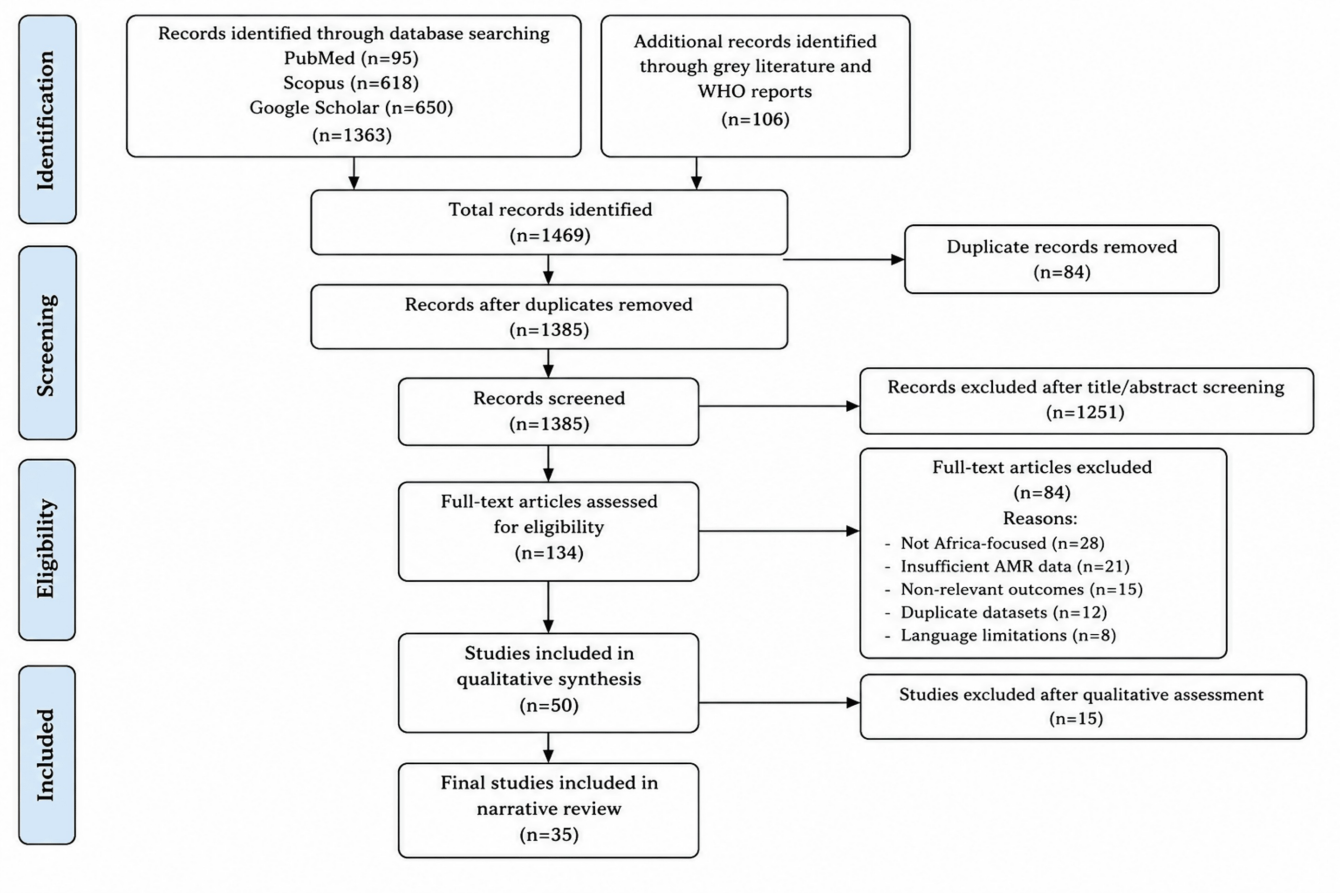
Literature identification and selection process used to support the narrative review (2014-2025)

To inform this narrative review, a comprehensive literature search was conducted using PubMed/MEDLINE, Scopus, and Google Scholar. Additional references were identified through manual screening of reference lists from relevant publications, WHO documents, international surveillance reports, and public health policy papers. Reports from major organizations involved in AMR surveillance and policy development, including the WHO, the Global Research on Antimicrobial Resistance (GRAM) project, and international public health initiatives, were also reviewed to complement peer-reviewed evidence [2-6]. The literature search was conducted between October and December 2025, with the final search completed in December 2025 (Figure 1).

The search strategy combined Medical Subject Headings (MeSH) and free-text terms related to AMR and Africa. Search terms included “antimicrobial resistance,” “antibiotic resistance,” “drug resistance,” “multidrug resistant,” “MDR,” “ESBL,” “MRSA,” “carbapenem resistance,” “antimicrobial stewardship,” “surveillance,” “antibiotic consumption,” “One Health,” and “infection prevention and control,” combined with geographic terms including “Africa,” “Sub-Saharan Africa,” “North Africa,” and “Morocco.” An example of a PubMed search query is as follows: (“antimicrobial resistance”[Title/Abstract] OR “antibiotic resistance”[Title/Abstract]) AND (Africa[Title/Abstract] OR “Sub-Saharan Africa”[Title/Abstract] OR Morocco[Title/Abstract]). Priority was given to peer-reviewed epidemiological studies, surveillance reports, systematic and narrative reviews, and publications from international organizations with direct relevance to AMR burden, surveillance systems, antimicrobial stewardship, and public health policy in Africa.

The review focused on publications addressing major public health dimensions of AMR in Africa, including epidemiological burden, surveillance systems, laboratory infrastructure, AST, antimicrobial stewardship, healthcare-associated infections, antibiotic misuse, and policy interventions [2-15]. Additional emphasis was placed on publications exploring One Health approaches, carbapenem resistance, multidrug-resistant gram-negative bacteria, and resistance trends among the WHO priority pathogens [16-22].

Eligibility Criteria

Publications were considered eligible if they addressed AMR in African countries, including North Africa and Sub-Saharan Africa, and if they included epidemiological findings, AMR surveillance data, resistance patterns in priority pathogens, antibiotic use practices, stewardship interventions, laboratory capacity issues, infection prevention and control measures, or policy responses related to AMR. Studies published in English or French between January 2014 and December 2025 were considered eligible for inclusion.

We included original research articles, epidemiological studies, systematic reviews, narrative reviews, multicenter surveillance studies, and reports from international organizations such as the WHO and other relevant institutions. Priority was given to studies with clear relevance to public health implications, surveillance systems, resistance burden, or antimicrobial policy strategies in African settings [2-7,11-18]. Publications specifically addressing carbapenem-resistant *Enterobacteriaceae*, multidrug-resistant *Acinetobacter baumannii*, ESBL-producing *Enterobacterales*, and stewardship challenges in low- and middle-income countries were also prioritized because of their increasing clinical and epidemiological importance [19-24].

Case reports, conference abstracts, conference proceedings, editorials without substantial epidemiological contribution, studies unrelated to Africa, and publications not directly addressing AMR were excluded. Although a transparent literature identification and selection process was conducted and summarized in Figure 1 to enhance transparency and comprehensiveness, this work remained intentionally designed as a narrative review rather than a formal systematic review. The flowchart was included solely to improve transparency regarding literature identification and selection and should not be interpreted as indicating adherence to the Preferred Reporting Items for Systematic reviews and Meta-Analyses (PRISMA) standards for systematic reviews. Consequently, no quantitative quality scoring system or formal risk-of-bias assessment was performed.

Study Selection

Retrieved publications were reviewed for relevance to the objectives of this narrative review. When necessary, full-text articles were examined to determine their contribution to the thematic synthesis. Full-text articles were reviewed whenever additional clarification regarding eligibility or relevance was required. Studies meeting predefined eligibility criteria were retained for synthesis. International reports and policy documents were included to complement peer-reviewed evidence, particularly in contexts where surveillance data remained fragmented or insufficiently represented in indexed literature [4-7,11,12].

Special attention was given to studies providing quantitative epidemiological data on AMR burden, resistance patterns among WHO priority pathogens, implementation of WHO-GLASS recommendations, stewardship interventions, or public health implications in African healthcare systems. In addition, Moroccan studies reporting resistance profiles among *Enterobacterales*, *Escherichia coli*, *Klebsiella pneumoniae*, *Acinetobacter baumannii*, and other clinically relevant pathogens were carefully reviewed because of their relevance to the Moroccan case study component of this review [4,23-27]. Several of the pathogens prioritized in this review, including* Escherichia coli *and *Klebsiella pneumoniae*, have been identified among the major contributors to the global AMR burden [28]. These findings are particularly relevant in developing countries, where inappropriate antibiotic use, limited diagnostic capacity, and inadequate infection control measures continue to accelerate the emergence and spread of AMR [29].

Data Extraction and Synthesis

Data extraction was performed using a standardized narrative approach focusing on epidemiological burden, resistance patterns, surveillance characteristics, stewardship dimensions, laboratory capacity, and public health implications. Extracted variables included geographic setting, study population, healthcare setting (community versus hospital), targeted pathogens, resistance mechanisms, antimicrobial susceptibility profiles, and policy or stewardship-related findings.

Particular attention was paid to resistance patterns involving methicillin-resistant *Staphylococcus aureus* (MRSA), ESBL-producing *Enterobacterales*, carbapenem-resistant gram-negative bacteria, and multidrug-resistant *Acinetobacter baumannii *because of their major implications for therapeutic failure, healthcare-associated infections, and intensive care unit management [19-24]. Information related to antibiotic misuse, self-medication, empirical broad-spectrum prescribing, inappropriate antibiotic dispensing, limited diagnostic infrastructure, and insufficient infection prevention and control measures was also systematically reviewed because these determinants are consistently identified as major drivers of AMR expansion in African settings [8-15,30-34].

Additional data regarding antimicrobial stewardship programs, surveillance implementation, laboratory strengthening, AST access, and One Health approaches were extracted to better understand structural barriers and opportunities for AMR mitigation in Africa [16-18]. Particular emphasis was placed on studies evaluating implementation challenges related to WHO-GLASS surveillance systems, quality assurance limitations, and integration of surveillance outputs into national public health policies [4-7].

The extracted evidence was then narratively synthesized and organized into major thematic domains including: (i) epidemiological burden and mortality associated with AMR, (ii) surveillance gaps and infrastructure limitations, (iii) determinants and drivers of resistance in community and healthcare settings, (iv) antimicrobial stewardship and infection prevention strategies, (v) public health policy responses and One Health approaches, and (vi) (vi) Morocco as an illustrative case study and source of contextual insights for African health systems. Because of the substantial heterogeneity of included studies in terms of design, populations, settings, outcome measurements, and surveillance methodologies, a quantitative meta-analysis was not performed.

Discussion

This review synthesizes current evidence on AMR in Africa and highlights major public health challenges while drawing lessons from Morocco as a relevant case study. Overall, the findings support three major observations: first, AMR represents a substantial and unevenly distributed public health burden across African regions; second, the drivers of AMR are largely convergent across community and healthcare settings; and third, surveillance systems remain fragmented because of heterogeneous implementation of WHO-GLASS recommendations and persistent structural limitations affecting laboratory and reporting capacity [4-7].

AMR as a Major and Disproportionate Burden in Africa

The available evidence confirms that AMR has become one of the most serious public health threats affecting African healthcare systems. Data from the GRAM project estimated that in 2019, approximately 4.95 million deaths were associated with bacterial AMR globally, including nearly 1.27 million deaths directly attributable to resistant bacterial infections [28]. Importantly, western Sub-Saharan Africa was reported to have the highest all-age AMR-attributable mortality rate worldwide, reaching 27.3 deaths per 100,000 population [29]. These findings illustrate the disproportionate burden affecting resource-constrained settings characterized by high infectious disease prevalence, delayed access to appropriate care, limited diagnostic infrastructure, and barriers to optimized antimicrobial therapy [5,29].

Future projections further reinforce the urgency of immediate intervention. Recent GRAM forecasts suggest that AMR-related mortality could increase substantially by 2050 if current trends persist, particularly in low- and middle-income countries where healthcare systems continue to face important structural limitations [5]. These observations are consistent with previous Africa-focused analyses emphasizing that AMR is not solely a microbiological issue but also a broader health system challenge involving inequities in healthcare access, laboratory capacity, infection prevention, and antimicrobial stewardship implementation [9-12]. The major epidemiological findings, surveillance gaps, and public health implications identified in this review are summarized in Table 1.

**Table 1:** Summary of major epidemiological findings, surveillance challenges, and public health implications of AMR in Africa and selected published evidence from Morocco AMR: antimicrobial resistance; GRAM: Global Research on Antimicrobial Resistance; GLASS: Global Antimicrobial Resistance Surveillance System; AST: antimicrobial susceptibility testing; ICU: intensive care unit; *E. coli: Escherichia coli; A. baumannii: Acinetobacter baumannii; K. pneumoniae: Klebsiella pneumoniae*

Domain	Indicator/pathogen	Key result	Public health interpretation
Global burden (GRAM)	AMR-associated deaths (2019)	4.95 million deaths associated with bacterial AMR [28]	Confirms AMR as a major global cause of mortality requiring urgent response
Global burden (GRAM)	AMR-attributable deaths (2019)	1.27 million deaths directly attributable to AMR [28]	Reflects direct excess deaths due to resistance-related treatment failure
Africa burden (GRAM)	Highest AMR-attributable death rate	Western Sub-Saharan Africa: 27.3 per 100,000 [28]	Suggests disproportionate burden in African regions with constrained health systems
Africa surveillance	WHO-GLASS implementation	Variable adoption across Africa [7]	Limits the comparability of AMR data, weakens evidence-based continental planning
Africa drivers	Antibiotic use & diagnostics	Empirical therapy + limited AST capacity [3]	Promotes broad-spectrum exposure and delayed targeted therapy
Morocco case study	*E. coli *resistance to amoxicillin	90.9% (median) [4]	Indicates very limited effectiveness of common first-line agents
Morocco case study	*E. coli* resistance to amoxicillin–clavulanic acid	64.0% (median) [4]	Suggests risk of therapeutic failure for common infections
Morocco case study	*E. coli *resistance to co-trimoxazole	56.0% (median) [4]	Supports revision of prescribing guidance and stewardship measures
Morocco case study	*K. pneumoniae *resistance to amoxicillin–clavulanic acid	63.0% (median) [4]	Highlights concern for severe infections requiring early effective coverage
Morocco case study	*A. baumannii* resistance to imipenem	74.5% (median) [4]	Suggests circulation of carbapenem-resistant strains in hospitals/ICUs
Morocco case study	Colistin resistance	0.1% (median) [4]	Colistin remains largely preserved → requires strict protection policies

Moroccan resistance estimates presented in this table are derived from published studies synthesized in the scoping review by Nejjari et al. and should not be interpreted as current national surveillance estimates. Variations in study settings, sampling strategies, time periods, and laboratory methodologies may affect comparability.

Drivers of AMR Across Community and Healthcare Settings

Our narrative synthesis indicates that AMR in Africa is sustained by interconnected drivers operating simultaneously in both community and healthcare environments. At the community level, inappropriate antibiotic exposure remains widespread because of non-prescription antibiotic access, self-medication, incomplete adherence, inappropriate dosing, and limited health literacy regarding antimicrobial use [3,10,11]. These practices contribute to continuous selective pressure favoring the emergence and dissemination of resistant microorganisms.

Within healthcare facilities, empirical broad-spectrum antibiotic prescribing is frequently unavoidable because of insufficient microbiological infrastructure and limited access to AST [3,6]. Delayed laboratory confirmation and restricted diagnostic capacity contribute to prolonged empirical therapy and inappropriate antimicrobial exposure, particularly in tertiary hospitals and intensive care units. In addition, inadequate IPC measures may facilitate transmission of multidrug-resistant organisms in healthcare environments [3-6].

These convergent pressures promote the persistence of clinically important resistant pathogens, including ESBL-producing Enterobacterales, MRSA, carbapenem-resistant *Enterobacteriaceae*, and multidrug-resistant *Acinetobacter baumannii *[12-14,19-22]. The increasing emergence of carbapenem resistance is particularly concerning because it significantly reduces therapeutic options for severe healthcare-associated infections and may increase mortality risk in vulnerable hospitalized patients [19-22].

Surveillance Systems: Progress and Persistent Structural Gaps

Effective surveillance remains essential for evidence-based AMR response, treatment guideline adaptation, and evaluation of national action plans. However, our review highlights that African countries continue to face major challenges related to surveillance standardization, laboratory capacity, AST implementation, and reporting completeness [6,7]. WHO-GLASS reports continue to demonstrate heterogeneous implementation across African settings, limiting comparability of AMR indicators between countries and weakening regional epidemiological coordination [3,7].

Several structural barriers continue to affect surveillance performance, including fragile laboratory infrastructure, shortages of trained microbiologists, inconsistent quality assurance systems, limited funding, and incomplete integration of surveillance outputs into national health information systems [7,12]. Consequently, AMR data remain fragmented and unevenly distributed, potentially underestimating the true epidemiological burden in several regions.

Another important limitation is that available AMR evidence often originates from tertiary hospitals and urban referral centers, potentially overrepresenting healthcare-associated resistance while underrepresenting community AMR dynamics in rural or underserved settings. Strengthening surveillance, therefore, requires investment not only in reference laboratories but also in decentralized laboratory networks, workforce development, quality management systems, and sustainable national reporting mechanisms [12,13].

Morocco as a Relevant African Case Study

The Moroccan case study reflects several of the structural determinants and epidemiological challenges observed more broadly across African healthcare systems. Published epidemiological evidence has documented high resistance levels among WHO priority pathogens, particularly *Escherichia coli*, *Klebsiella pneumoniae*, and *Acinetobacter baumannii *[4,27-29]. These findings should be interpreted as evidence derived from published studies rather than nationally representative surveillance estimates, as available data originate from heterogeneous institutions, populations, study periods, and laboratory settings. The scoping review conducted by Nejjari et al. remains one of the most comprehensive syntheses of AMR evidence available in Morocco and highlights substantial resistance rates to commonly used antibiotics, including amoxicillin, amoxicillin-clavulanic acid, and co-trimoxazole [4].

Particularly concerning is the high median resistance to imipenem observed in *Acinetobacter baumannii*, suggesting circulation of multidrug-resistant and carbapenem-resistant strains in Moroccan healthcare facilities, especially in intensive care units and high-risk hospital departments [4]. These findings align with broader African evidence documenting the emergence of carbapenem resistance and increasing prevalence of multidrug-resistant gram-negative bacteria in healthcare-associated infections [19-22].

At the same time, Morocco illustrates both progress and persistent limitations in AMR response. Although initiatives related to antibiotic regulation, surveillance strengthening, and public awareness have been implemented, national surveillance outputs remain heterogeneous, and evaluation of intervention effectiveness remains limited [1,6-8]. Morocco, therefore, provides valuable transferable lessons regarding the importance of linking surveillance systems to stewardship interventions, IPC programs, and evidence-informed policy implementation.

Public Health Implications and Strategic Priorities

Our findings support prioritizing interventions that are feasible, scalable, and adapted to resource-constrained settings. Strengthening laboratory diagnostic capacity and improving access to AST remain essential to reduce unnecessary broad-spectrum prescribing and facilitate targeted antimicrobial therapy [12,13]. Similarly, scaling up IPC programs in healthcare facilities represents a cost-effective strategy to reduce transmission of resistant organisms, particularly in high-risk wards such as intensive care units [17].

Antimicrobial stewardship programs should progressively be integrated into routine clinical practice using locally adapted prescribing guidelines informed by surveillance data, audit and feedback mechanisms, and restriction policies for reserve antibiotics [15,16]. At the community level, strengthening regulation of nonprescription antibiotic dispensing and promoting sustained public awareness campaigns remain essential to reduce misuse and inappropriate antimicrobial exposure [1,3,10].

The One Health approach also remains central to the African context because antimicrobial use in humans, livestock, agriculture, and environmental systems may collectively contribute to resistance selection and dissemination [9,17]. Effective AMR response, therefore, requires multisectoral governance, long-term political commitment, and regional collaboration integrating human health, veterinary medicine, food production, and environmental surveillance systems.

Strengths and limitations

This review provides a policy-oriented synthesis of current evidence regarding AMR burden, surveillance gaps, and public health implications in Africa while integrating Morocco as a relevant case study. However, several limitations should be acknowledged. First, this work was intentionally designed as a narrative review and therefore does not follow the methodological rigor of a formal systematic review or meta-analysis. No formal risk-of-bias assessment or quantitative quality scoring system was performed. The literature search and selection process was used to improve transparency and comprehensiveness of the evidence synthesis, but was not intended to fulfill all methodological requirements of a formal systematic review.

Second, available evidence remains highly heterogeneous in terms of study design, surveillance methodologies, healthcare settings, and outcome measurements. In addition, several African countries continue to have limited publication output and incomplete surveillance coverage, which may lead to underrepresentation of local AMR dynamics [7]. Furthermore, differences in laboratory methodologies, AST standards, and reporting practices across studies may have influenced the comparability of resistance estimates between settings. Finally, Moroccan estimates were largely synthesized from a single comprehensive scoping review, which may not fully capture recent unpublished institutional surveillance data or regional variations [4]. Consequently, the reported resistance estimates should be interpreted with caution and should not be considered nationally representative surveillance figures. Nevertheless, by triangulating peer-reviewed literature, surveillance reports, and international public health evidence, this review provides a broad and policy-relevant overview of the major AMR challenges currently affecting African healthcare systems.

## Conclusions

To conclude, our narrative review indicates that AMR in Africa has become a critical public health threat, driven by interconnected determinants across both community and healthcare settings. The evidence summarized in this review highlights the combined effect of inappropriate antibiotic use, nonprescription access in many contexts, persistent reliance on empirical broad-spectrum therapy due to limited diagnostic capacity, and insufficient infection prevention and control measures. These challenges are further compounded by major surveillance gaps and heterogeneous implementation of the WHO-GLASS recommendations, resulting in fragmented and often noncomparable data across countries. Strengthening AMR surveillance, laboratory capacity, antimicrobial stewardship, and coordinated One Health policies remains essential to reduce avoidable morbidity and mortality and to preserve the effectiveness of existing antibiotics. Without urgent and coordinated action, AMR will continue to erode progress in infectious disease control and threaten health system resilience across the continent.

Morocco, which we presented as a case study, illustrates many of these continental challenges while also providing useful lessons for public health action. Available evidence indicates high resistance rates among priority pathogens, particularly *Enterobacterales *(*Escherichia coli *and *Klebsiella pneumoniae*) and carbapenem-resistant *Acinetobacter baumannii, *which threaten the effectiveness of commonly used first-line treatments and increase reliance on last-resort antibiotics. At the same time, Morocco has initiated efforts aligned with international recommendations to improve regulation, surveillance, and awareness. Morocco’s experience suggests that strengthening surveillance is necessary but not sufficient and must be implemented alongside effective stewardship and robust IPC programs in order to translate data into measurable public health impact. Consolidating these initiatives through sustained investment in laboratory networks, standardized reporting, stewardship implementation, and effective IPC programs would not only improve national outcomes but also contribute to a stronger regional AMR control framework.
